# Improving Interdisciplinary Working Between Psychiatry Resident Doctors and Nurses in Lancashire and South Cumbria NHS Foundation Trust – A Quality Improvement Project

**DOI:** 10.1192/bjo.2026.11453

**Published:** 2026-06-30

**Authors:** Babatunde Oyesakin, Stef Osborn

**Affiliations:** 1 Lancashire and South Cumbria NHS Foundation Trust, Preston, United Kingdom; 2 Lancashire and South Cumbria NHS Foundation Trust, Cumbria, United Kingdom

## Abstract

**Aims:**

To identify themes that underlie fractures in the relationship between doctors and nurses in the trust and recommend interventions to help improve the situation.

**Methods:**

Incident reports relating to difficult working relationship between resident doctors and nurses over 12 months were examined. The recurrent themes were identified, and thematic analysis was done to group them according to locality and main issues involved. Locality of focus was The Harbour inpatient complex.

Questionnaires were administered to nursing staff on duty on the Harbour inpatient units to resident doctors who had been on call at The Harbour in the previous 12 months. The answers were analysed, and the most common themes were identified and developed into recommendations.

**Results:**

A total of 18 incident reports were received in the trust in the year under review which involved doctors making reports about nurses and/or vice-versa.

The themes identified were:

(A) Issues with communication such as aggressive tone, poor bedside manner.

(B) Doctors being unavailable or non-contactable such as busy with other duties, asleep.

(C) Issues with clinical decision-making such as disagreeing over patient management decisions.

(D) Poor handover such as lacking important information, requests to review considered inappropriate for an on-call situation.

Nursing questionnaire results: 21 respondents raised similar issues as above (7 respondents raised Issue A, 9 issue B, 6 issue C and 4 respondents had other issues).

Doctors’ questionnaire results: 10 respondents raised issue C, 8 raised issue D, 2 raised all issues and 2 had other issues.

Recommendations made include:

- Nurses’ Training in handover to be in the SBAR format – Situation, Background, Assessment and Recommendation, teaching given on how to escalate differences in clinical opinion to senior medics on call.
- Doctors advised on how to manage night shifts as regards rest times, discuss requests to review and how to be more visible on the wards.

Follow-up work is currently on-going to determine the impact of the recommendations. Preliminary reports show a reduction in the number of incident reports relating to fractious working relationship between doctors and nurses.

**Conclusion:**

There is potential for friction in any working relationship, particularly in the context of a highly pressurised working environment such as acute mental health units, probably worsened by understaffing, presence of bank staff who may be unfamiliar with the patients and other interpersonal factors.

We conclude that relatively simple interventions such as handover training, shift management and education on proper use of incident reporting systems may help alleviate such fractures and improve working relationship.

